# A standardized method for lectin microarray-based tissue glycome mapping

**DOI:** 10.1038/srep43560

**Published:** 2017-03-06

**Authors:** Xia Zou, Maki Yoshida, Chiaki Nagai-Okatani, Jun Iwaki, Atsushi Matsuda, Binbin Tan, Kozue Hagiwara, Takashi Sato, Yoko Itakura, Erika Noro, Hiroyuki Kaji, Masashi Toyoda, Yan Zhang, Hisashi Narimatsu, Atsushi Kuno

**Affiliations:** 1 Biotechnology Research Institute for Drug Discovery, National Institute of Advanced Industrial Science and Technology (AIST), Tsukuba 305-8568, Japan; 2 Ministry of Education, Key Laboratory of Systems Biomedicine, Shanghai Center for Systems Biomedicine, Shanghai Jiao Tong University, Shanghai 200240, China; 3 Research Team for Geriatric Medicine, Tokyo Metropolitan Institute of Gerontology, Tokyo 173-0015, Japan

## Abstract

The significance of glycomic profiling has been highlighted by recent findings that structural changes of glycans are observed in many diseases, including cancer. Therefore, glycomic profiling of the whole body (glycome mapping) under different physiopathological states may contribute to the discovery of reliable biomarkers with disease-specific alterations. To achieve this, standardization of high-throughput and in-depth analysis of tissue glycome mapping is needed. However, this is a great challenge due to the lack of analytical methodology for glycans on small amounts of endogenous glycoproteins. Here, we established a standardized method of lectin-assisted tissue glycome mapping. Formalin-fixed, paraffin-embedded tissue sections were prepared from brain, liver, kidney, spleen, and testis of two C57BL/6J mice. In total, 190 size-adjusted fragments with different morphology were serially collected from each tissue by laser microdissection and subjected to lectin microarray analysis. The results and subsequent histochemical analysis with selected lectins were highly consistent with previous reports of mass spectrometry-based *N*- and/or *O*-glycome analyses and histochemistry. This is the first report to look at both *N*- and *O*-glycome profiles of various regions within tissue sections of five different organs. This simple and reproducible mapping approach is also applicable to various disease model mice to facilitate disease-related biomarker discovery.

As one of the most common and fundamental post-translational modifications, glycosylation plays essential roles in almost every biological process, including cell differentiation and proliferation, cell-cell and cell-matrix interactions, and immune responses^1^,^2^. Glycosylation is highly heterogeneous with site-, protein-, cell-, and tissue-specific expressions^3^, which make it possible to reflect the changes in various physiopathological states of the cell, i.e., the origin and the developmental stage of tissue, and the presence and extent of malignancy^4^. In fact, aberrant glycosylations have been widely observed in many human diseases including cancer^3^, autoimmune disease^5^,^6^, and inflammatory responses^7^. For instance, significant increases in α2,6-sialylation and core fucosylation have been reported in various malignancies including colon^8^, stomach^9^, lung^10^, and breast cancer^11^. In particular, the glycan antigen sialyl-Le^a^ (CA 19-9) and core fucosylation of α-fetoprotein (AFP-L3) are cancer-associated markers widely used in clinical practice^12^,^13^. Additionally, increased GlcNAc-branching *N*-glycan expression and the overexpression of truncated *O*-glycans are also common features of tumors^3^,^14^,^15^. Therefore, glycomic profiling of tissues under different physiopathological states would provide a source of reliable tissue-specific biomarkers with disease-specific alterations and a set of targets for therapeutic intervention, as well as provide further insight into understanding the mechanisms of glycan-mediated biological events and diseases.

Over the past decade, mass spectrometry (MS)-based approaches, including liquid chromatography (LC)-MS, have been developed as the most common strategies for glycomic analysis^16^ and have been applied to tissue specimens^17^,^18^,^19^. Traditionally, these strategies have mainly focused on profiling the total glycans enzymatically or chemically released from proteins, which has the advantage of allowing detailed glycan structural analysis with a high degree of accuracy, but loses information on the proteins that carry the glycans^20^. Another approach is direct analysis of glycopeptides without releasing the glycans from the peptides, which is achieved by tandem mass spectrometric analysis using high-energy collision-induced dissociation or electron-transfer dissociation MS^21^. There has been continued progress in the discrimination of structural, linkage, and positional isomers of glycans as well as quantification of *N*-glycans^22^,^23^,^24^, though these methods are typically time-consuming and require extensive sample preparation procedures^25^,^26^. In recent years, the assessment of glycan changes in tissues based on matrix-assisted laser desorption/ionization imaging MS (MALDI-IMS) has gained increasing attention. This technique provides direct visualization and evaluation of *N-*glycans in different pathological regions of fresh/frozen tissues^27^ and formalin-fixed paraffin-embedded (FFPE) tissue sections^28^,^29^,^30^,^31^. This approach is the best fit to analyze released free glycans with relatively complex sample preparation^32^.

Compared with the technologies described above, lectin microarray is a simple and highly sensitive method to directly obtain global glycomic profiling of *N*- and *O*-glycans in both pure and crude glycoprotein samples without the need to release or purify glycan moieties or other complex preparation procedures^25^. From a biological point of view, the use of lectin microarray for comparative analysis potentially contributes to differential glycoproteomics, because the glycoproteins are almost in their intact forms without peptide fragmentation or glycan liberation, which maintains their density and natural orientation. Such features make lectin microarrays suitable for the initial detection of glycome differences in biological samples, although it yields less detailed structural information. Currently, lectin microarray has proven useful in assessing the characteristics of tumors and for screening novel biomarkers for cancer diagnosis^33^. Based on highly sensitive evanescent-field fluorescence-assisted lectin microarray^34^, we have established a feasible method for differential glycan profiling targeting very small regions (i.e., “one-dot” sections comprising about 1000 cells) on FFPE tissue sections after delipidation^35^,^36^, and identified *Wisteria floribunda* agglutinin (WFA) as the most reliable lectin probe to detect cholangiocarcinoma-specific glyco-alterations^37^. Alternatively, lectin microarrays have been used to select the best lectin probe as a sensitive imaging molecule to identify high-grade dysplastic lesions of tissue, which compensates for the limitations of conventional endoscopy^38^. For further application, the technology should be applied to tissue glycome mapping, a new technique to collect and observe the glycomes of serial fragments in a tissue section derived from different organs. To be used for the whole body^39^, the method should be standardized for continuous data accumulation. In this study, we report an improved strategy integrating ultrasensitive techniques, namely laser microdissection (LMD)^40^ for tissue dissection and lectin microarray for glycan analysis.

## Results

### Pilot experiment for mouse tissue glycome mapping using lectin microarray with manual dissection

For mouse tissue glycomic analysis by means of lectin microarray, we initially conducted a pilot experiment targeting 94 FFPE tissue fragments (~1.0 mm^2^) conventionally dissected by hand under a microscope from a commercialized tissue array comprising 11 mouse organs (brain, lung, heart, thymus, spleen, pancreas, skin, kidney, small intestine, testis, and liver) (Supplementary Figure S1a). Hierarchical clustering (HC) analysis using the obtained 94 glycomic profiles provided a heat map, which clearly showed that different organs could be basically distinguished according to their characteristic glycan profiles (Supplementary Figure S1b). However, in this case, the appropriate scanning gain conditions with net intensities of all positive spots <40,000 for each sample from one organ varied a lot (Supplementary Figure S2a). We speculate that these notable signal variations were mainly attributable to the manual dissection process, which resulted in frequent sampling errors and large size variations, and thus caused time-consuming data normalization and relatively inaccurate statistical analysis. Given this, the conventional manual dissection process is not suitable for data accumulation aimed at comprehensive glycome mapping for whole organs.

### Improved data accumulation of lectin microarray by combination with LMD

**Figure 1:**
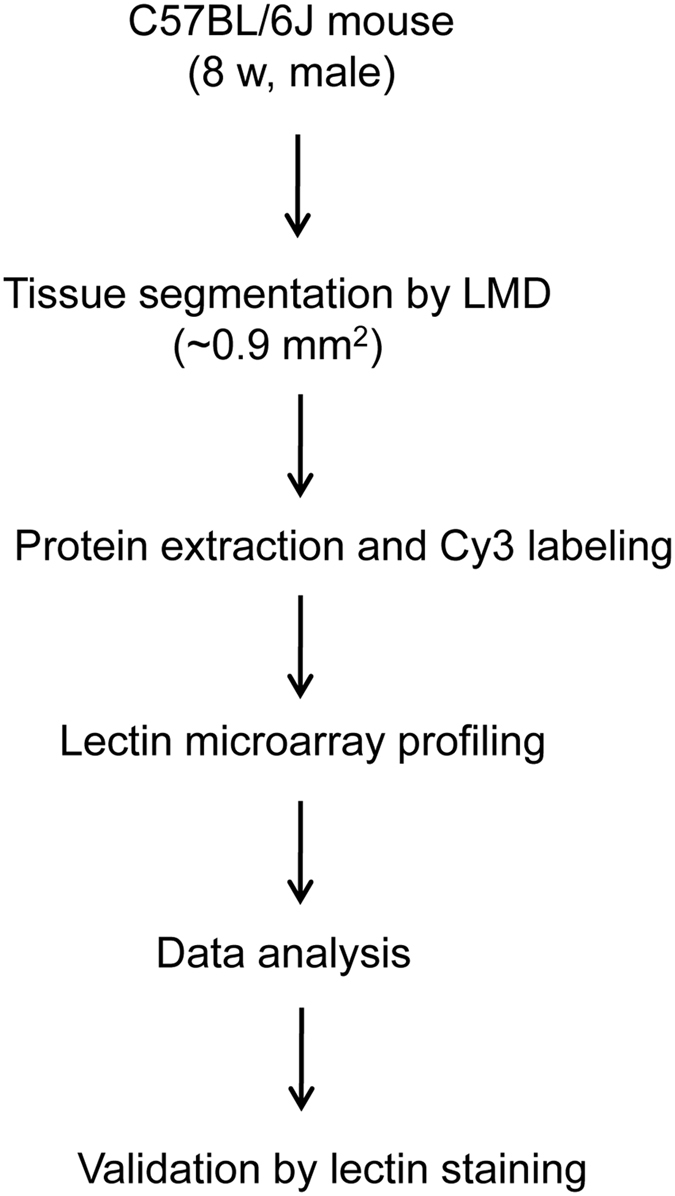
Scheme of tissue glycomic profiling using LMD and lectin microarray. Approximately 0.9 mm^2^ of tissue fragments (5 μm thickness) were collected from FFPE tissue sections of five mouse organs (brain, liver, kidney, spleen, and testis) by LMD. Proteins were extracted, Cy3-labeled, and subjected to lectin microarray analysis. The signal patterns of lectins were selectively verified using lectin staining.

To eliminate the variation from manual dissection, we next introduced LMD as an ultrasensitive technique for tissue dissection. The improved standard strategy combining LMD with lectin microarray for tissue glycome mapping is summarized in Fig. 1. Briefly, small FFPE tissue fragments (0.900 to 0.918 mm^2^ and 5 μm thickness) of five organs (brain, liver, kidney, spleen, and testis) from Mouse 2 and 3 were collected by LMD. After protein extraction and Cy3-labeling, the samples were subjected to lectin microarray analysis and the specific signal patterns were selected and verified using lectin staining. For the initial analysis, tissue fragments of five organs (19 fragments of brain, 6 of liver, 13 of kidney, 4 of spleen, and 6 of testis) were collected by LMD depending on histomorphological differences (Supplementary Figure S3) and were subjected to lectin microarray. Representative lectin profiling images of five organs are shown (Supplementary Figures S4 and S5). Importantly, this strategy improved the appropriate scanning gain conditions for each tissue section from one organ to the same level (Supplementary Figure S2b). In total, 190 tissue fragments were eventually collected from two mice, and 8 samples with low signal intensity or high noise level were excluded from the subsequent statistical analysis (Supplementary Figure S6). The normalized intensities of 45 lectins in the remaining 182 samples are shown in Supplementary Table S2.

### Comprehensive representation of differential *N-* and *O-*glycome profiles among five different organs

**Figure 2:**
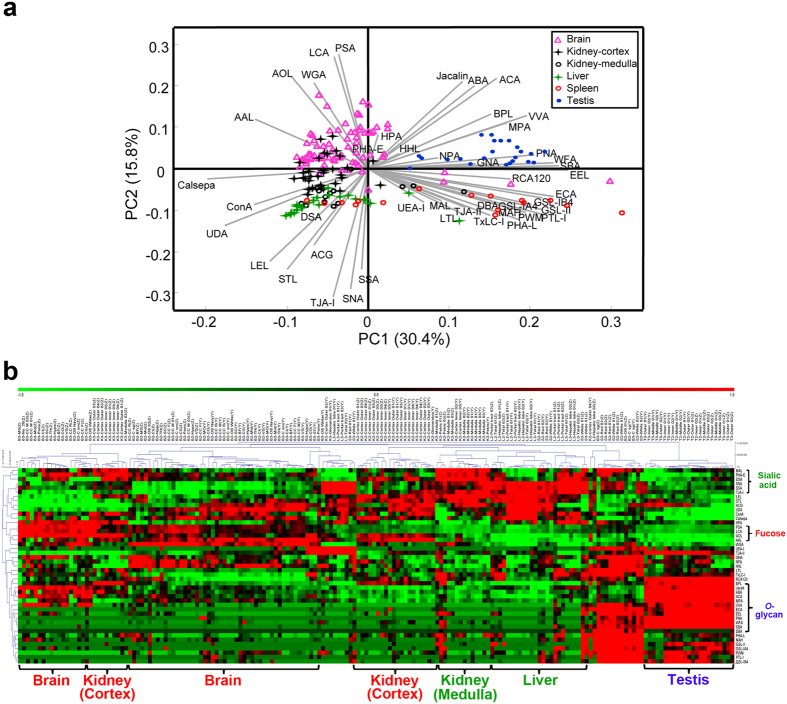
Differential glycomic profiling of tissue fragments obtained by LMD. (**a**) PCA of normalized lectin microarray data from the 182 tissue fragments of five organs (brain, liver, kidney, spleen, and testis) of Mouse 2 and 3. Each point represents one tissue fragment, and each of the 45 lectins is indicated by a vector shown in gray. The direction and length of a vector indicate how each lectin contributes to the two principal components for discriminating these tissue fragments, as labeled in the graph. (**b**) Two-dimensional HC analysis of the normalized lectin microarray data from the 182 tissue fragments. The 182 samples are listed in columns and the 45 lectins are listed in rows. The color and intensity of each square indicate the lectin signal levels in specific tissue fragments (Red, high; green, low; black, medium).

To compare the characteristics of both *N*- and *O*-glycome profiles among five different organs, the obtained tissue glycome profiles of the remaining 182 samples (73 fragments of brain, 24 of liver, 46 of kidney, 16 of spleen, and 23 of testis) were subjected to principal component analysis (PCA). These tissue fragments obtained from different organs could be divided into several groups by the signal patterns of 45 lectins (Fig. 2a). In particular, the fragments of testis showed the largest difference from the others, with 30.4% and 15.8% of the total variation for the first and second components, respectively. The distance biplot of PCA showed that the lectins recognizing core fucose, including AOL, AAL, LCA, and PSA, have relatively strong intensities in the fragments of the brain and cortex of kidney (Fig. 2a). The lectins recognizing terminal α2,6-linked sialic acid (SNA, SSA, and TJA-I) have relatively strong intensities in the fragments from liver and medulla of kidney according to HC (Fig. 2b). In addition, the lectins recognizing *O*-glycans such as Tn-antigen and T-antigen (PNA, WFA, MPA, and ACA) had relatively strong intensities from fragments of testis.

Next, *N*- and *O*-glycome profiles of the 182 tissue fragments were separately subjected to PCA and HC analysis (Supplementary Figure S7); the results of 33 lectins recognizing *N*-glycans (Supplementary Table S3) are shown in Figure S7a and c, whereas those of the 32 lectins recognizing *O-*glycans (Supplementary Table S3) are shown in Figure S7b and d. Unlike the glycomic profile using all the lectins (Fig. 2), the *N*-glycan profile of the brain samples showed the largest difference from the other organs, with higher signals of lectins recognizing fucose (AOL, AAL, LCA, and PSA) and mannose (ConA, Calsepa, GNA, NPA, and HHL). The *N*-glycan profiles of tissue fragments from testis and spleen sections showed similar patterns, with higher signals of the lectins recognizing GlcNAc (PWM), LacNAc (ECA), α-Gal (EEL), and GalNAc (WFA). By contrast, the *O*-glycan profile was more similar to the glycomic profile using all the lectins; the testis sections showed the highest signals of a panel of lectins recognizing *O*-glycans such as Jacalin, ABA, ACA, MPA, VVA, and PNA. The high intensities of lectins recognizing core fucose in the tissue fragments from brain and cortex of kidney, as well as the high intensities indicating high α2,6-sialylation in the samples of liver and medulla of kidney, were similar to the results using all the lectins.

### Differential glycomic profiling in a specific organ, the kidney

**Figure 3:**
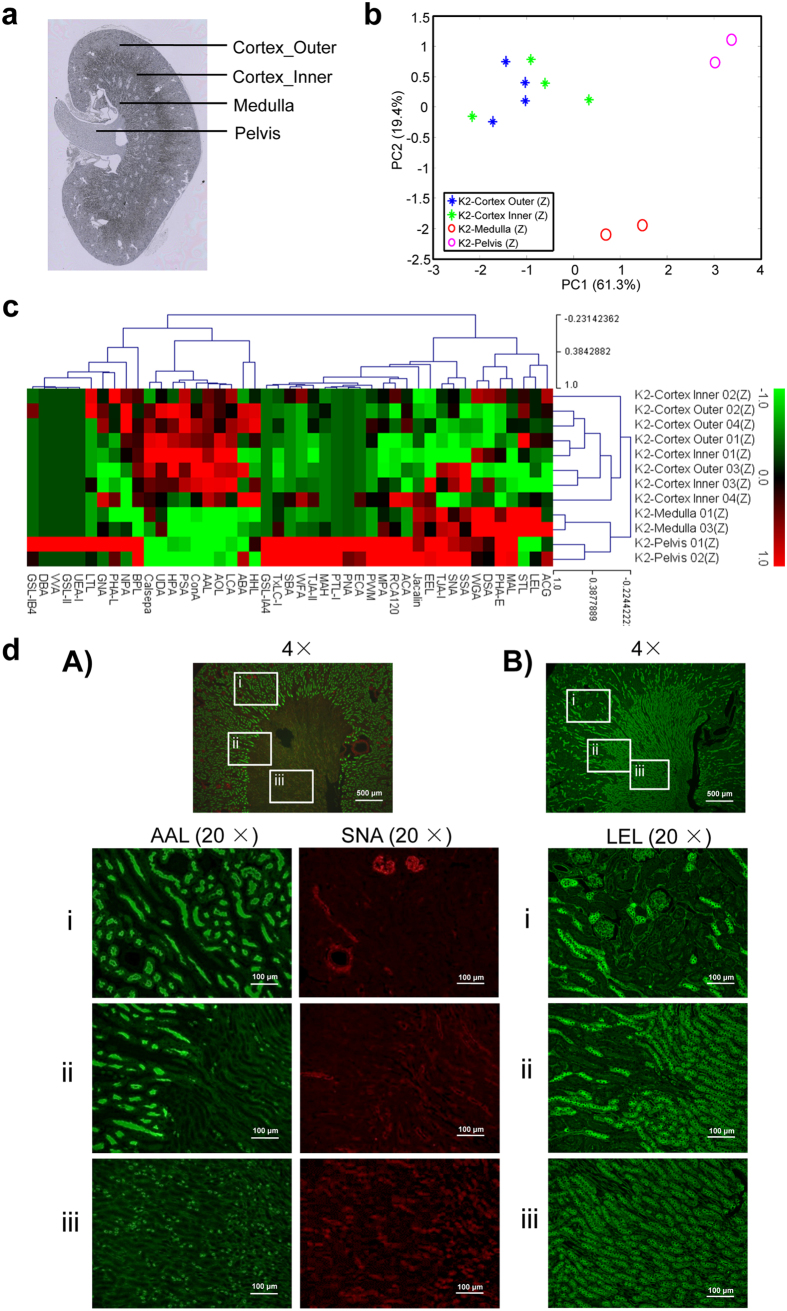
Differential glycomic profiling in kidney. (**a**) Hematoxylin-stained kidney section image annotated with the inner and outer cortex, medulla, and pelvic regions. (**b**) PCA of the normalized lectin microarray data on the tissue fragments from kidney of Mouse 2 (total 12 samples). Each point represents one tissue fragment. (**c**) Two-dimensional HC analysis of the lectin microarray data shown in (**b**). The 12 samples are listed in columns and the 45 lectins are listed in rows. The color and intensity of each square indicate the lectin signal levels in specific tissue fragments (Red, high; green, low; black, medium). (**d**) Histochemical validation of differential glycome profiling in kidney by double staining with AAL recognizing fucose (green) and SNA recognizing α2,6-linked sialic acid (red) (A), and by staining with LEL recognizing GlcNAc oligomer (green) (B). The enlarged images of three positions in cortex (i), cortex-medulla interface (ii), and medulla (iii) are shown.

We next examined whether the lectin microarray data obtained as above can be applied to differential glycomic profiling in a specific organ. Because the morphologically different regions of kidney showed distinct signal patterns from 45 lectins, we further compared the glycan profiles in each region within the cortex, medulla, and pelvis (Fig. 3a). The PCA and HC results of 12 tissue fragments of Mouse 2 showed that the fragments of cortex were obviously distinguishable from those of medulla and pelvis (61.3% and 19.4% of the total variation for the first and second components, respectively) (Fig. 3b), with different signal patterns of lectins (Fig. 3c). Similar results were obtained from the other duplicates of kidneys from Mouse 2 and 3 (Supplementary Figure S8). The signals of 21 lectins were significantly different (*P* < 0.05) in the cortex and the other parts of the kidney (Table 1); in the cortex samples, the nine lectins showed higher signals, while the 12 lectins showed lower signals. Notably, the lectins showing the most significantly higher signals in the cortex were the ones recognizing fucose (AOL, AAL, LCA, and PSA) and GalNAc (BPL), while the lectins recognizing sialic acids (MAL, SNA, SSA, and TJA-I), T/Tn-antigen (MPA), and polylactosamine (LEL) showed the most significantly decreased signals in the cortex (Table 1).

Considering that the net signal intensities of BPL and MPA in kidney were relatively low (Supplementary Figure S5), we chose AAL, SNA, and LEL for further validation by lectin staining. AAL/SNA double staining and LEL staining of the kidney sections indicated that the fluorescence signal intensities of AAL were significantly high and those of SNA and LEL were significantly low in the cortex (Fig. 3d). The quantitative data showed 2- or 3-fold changes between samples collected from renal cortex and medulla, which is consistent with the lectin microarray results (Supplementary Figure S9) and previous reports on the localization of proteins with core and/or Lewis fucosylation on the surface of renal tubular cells in the cortex^41^,^42^.

### Differential glycomic profiling of a specific organ, the testis

**Figure 4:**
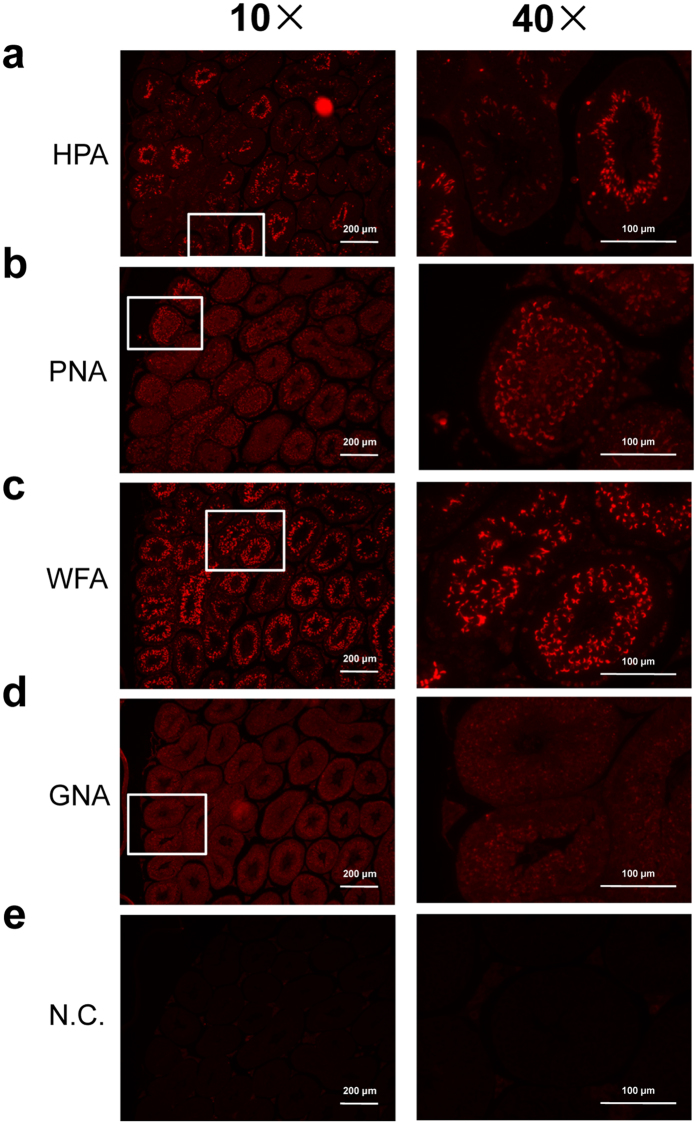
Histochemical validation of differential glycomic profiling in testis by lectin staining. The results of the lectin microarray were histochemically confirmed by lectin staining with (**a**) HPA recognizing α-linked terminal GalNAc residue (Tn-antigen), (**b**) PNA recognizing the Galβ1-3GalNAc structure (T-antigen), and (**c**) WFA recognizing both the terminal GalNAc residue and Galβ1-3GalNAc structure. (**d**) GNA recognizing high-mannose type *N*-glycan was used as a control. (**e**) PBS was used as the negative control (N.C.) instead of these lectins.

As the sections of testis showed the most significant difference from the other four organs (Fig. 2), we further verified the higher expression of *O*-glycans in the testis. For the lectin staining validation, we chose three representative lectins recognizing the typical structures of *O*-glycans, including HPA recognizing α-linked terminal GalNAc residues (Tn-antigen), PNA recognizing the Galβ1-3GalNAc structure (T-antigen), and WFA recognizing both the terminal GalNAc residue and Galβ1-3GalNAc structures. The lectin GNA, which recognizes the structure of high-mannose type *N*-glycans, was used as a control. The fluorescence signal intensities of HPA, PNA, and WFA were significantly higher in the testis compared with the signal of GNA staining (Fig. 4), confirming the results of the lectin microarray in which testis showed higher expression level of *O*-glycans but lower amounts of high-mannose type *N*-glycans.

**Figure 5:**
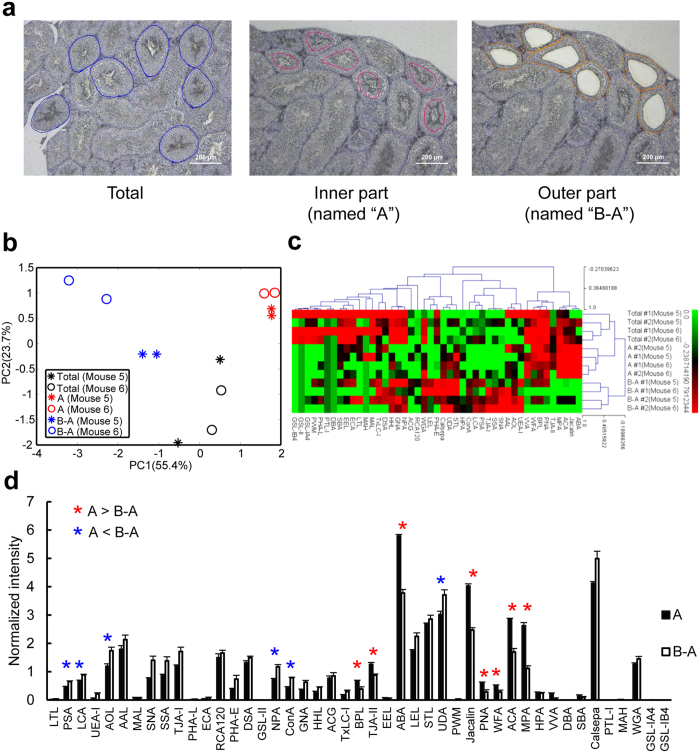
Differential glycomic profiling in morphologically different regions of seminiferous tubules. (**a**) Representative images of tissue dissection from the whole (named “total”), inner (“A”), and outer (“B-A”) parts of seminiferous tubules by LMD. (**b**) PCA of the normalized lectin microarray data from the 12 fragments from seminiferous tubules of Mouse 5 and 6. Each point represents one tissue section. (**c**) Two-dimensional HC analysis of the normalized lectin microarray data shown in (**b**). The 12 samples are listed in columns and the 45 lectins are listed in rows. The color and intensity of each square indicate the lectin signal levels in specific tissue fragments (Red, high; green, low; black, medium). (**d**) Comparison of the normalized data of the inner and outer parts. Asterisks indicate significant signals of lectins between these two parts (*P* < 0.05, paired *t*-test).

Notably, lectin staining showed that the localization of *O*-glycan binders was limited at the inner part of seminiferous tubules with different staining patterns (Fig. 4a–c). To elucidate the characteristics of the glycome in each part, tissue sections from the total, inner, and outer parts of the seminiferous tubules were collected by LMD, wherein the inner and outer parts were collected from identical tubules (Fig. 5a). In this study, a total of 12 tissue sections were obtained from two mice (Mouse 5 and 6), and each section was collected from 30 seminiferous tubules (Supplementary Figure S10). The PCA and HC analysis of the normalized lectin microarray data showed that the glycan profiles of the inner parts were obviously distinguishable from those of the outer parts (55.4% and 23.7% of the total variation for the first and second components, respectively) (Fig. 5b), with different signal patterns of lectins (Fig. 5c). The signals of eight lectins including *O*-glycan binders (Jacalin, PNA, WFA, ACA, MPA, and ABA) in the inner part were significantly higher than those in the outer part (Fig. 5d), which was consistent with the lectin staining pattern (Fig. 4). In addition, the analysis also identified lectins recognizing fucose (PSA, LCA, and AOL) as part-specific lectins in one seminiferous tubule (Fig. 5c). These results demonstrate that fragment dissection based on histochemistry using tissue-specific lectins selected by glycome mapping is an improved method to clarify the characteristics of a cell type-specific glycome.

## Discussion

In recent years, the significance of glycomic profiling has been highlighted by the fact that defects in glycosylation have been observed in many human diseases^43^,^44^. The investigation of whole tissue glycome mapping, i.e., comprehensive representation of sequential glycomic profiling of tissue sections derived from each organ, is a basic and open-ended research project but may provide key information to further understand various physiopathological events, and thus may be used to discover disease-related glycan alterations. Currently, MS-based glycan profiling using LC-MS and MALDI-IMS is a common glycomic analysis technique, but has limitations with parallel representation of both *N*- and *O*-glycomes as well as in throughput and reproducibility due to the complexity of sample preparation^22^,^32^. Thus, we aimed to establish a simple, standardized method for tissue glycome mapping by using a lectin microarray, which has been recognized as a sensitive and reliable tool for glycome analysis of tiny amounts of biological specimens^34^,^35^. We established an improved approach integrating LMD and a lectin microarray for glycomic analysis targeting restricted regions of FFPE tissue specimens, and demonstrated the feasibility of this approach in five organs (brain, liver, kidney, spleen, and testis) originating from C57BL/6J mice. Compared with other techniques for glycan profiling, our strategy has significant advantages in that: (1) an ideal combination of two ultrasensitive techniques enables glycan analysis for an extremely small volume of tissue comprising approximately 200 cells, (2) simple manipulation without the need for the release or purification of glycan moieties or other complex preparation procedures enables reproducible and high-throughput analysis for a large number of clinical specimens, (3) the 45-lectin microarray enables rapid and global glycomic profiling of both *N*- and *O*-glycans, (4) elimination of the variation resulting from manual dissection by using LMD enables more reliable analysis, and (5) semi-quantitative profiling data providing information on the distribution, predominance, and characteristic features of glyco-epitopes among the different tissue sections can be obtained. However, we should note that as lectins are of diverse specificity, some have cross-reactivity with various glycans. It is relatively difficult to characterize a specific glycan using only one lectin. Therefore, it is necessary to integrate the signals of several lectins with similar binding specificities, which is a relatively complicated interpretative process. If these lectins show a consistent change in pattern, we presume that the glycan profiles have changed. In addition, because of the cross-reactivity of lectins, we performed further immunohistochemical validation to confirm the signals of the lectin microarray before forming conclusions.

In this study, our approach successfully revealed tissue-specific glycome mapping in different organs. There is a report showing comprehensive and detailed comparison of glycan structures of mouse brain and liver tissues in a protein-specific and site-specific manner by LC-MS^45^. Nevertheless, as far as we know, this is the first report comparing tissue-specific glycosylations in as many as five organs at the same time. The finding of a higher level of fucosylation (recognized by AOL, AAL, LCA, PSA, *et al*.) in the brain and a higher level of sialylation (recognized by SNA, SSA, TJA-I, *et al*.) in the liver observed in our study (Fig. 2) was highly consistent with the results from the above study^45^. In addition, our results were also consistent with those by other groups surveying glycan pools derived from mouse brain or liver using different technologies. For example, Ji *et al*. found that the most abundant glycans observed in mouse brain were fucosylated complex/hybrid type glycans using a strategy of combining tissue glyco-capture and nano-LC-MS^19^. Eshghi *et al*. reported that more than 70% of all identified glycans appeared to be fucosylated from FFPE mouse brain tissue sections using MALDI-IMS^32^. With regard to glycomic analysis of the liver, a previous report using a lectin microarray and lectin staining revealed a positive signal from MAL-II recognizing sialylation in mouse liver, and the signal was increased on the surface of hepatocytes during the progression of fibrosis^46^. Another important feature of the tissue-specific glycans observed in our study is the higher level of *O*-glycans (recognized by HPA, PNA, WFA, *et al*.) in testis sections (Fig. 2), which was confirmed by lectin staining (Fig. 4) and is consistent with the following two recent studies. One study showed that the signal intensity of ABA and MPL recognizing *O*-glycans significantly decreased in sperm with a mutation in *DEFB126*, suggesting these lectins as potential biomarkers for clinical diagnosis of subfertility^47^; the other demonstrated that the level of *O*-glycans was related to the female-induced sperm acrosome reaction and surface carbohydrate reorganization^48^. It is interesting to note that the signals of Jacalin, ABA, ACA, MPA, and VVA were also high in the brain (Fig. 2b), which indicates a relatively high level of *O*-glycans in the brain as well as the testis. These results are consistent with the relatively higher expression of *O*-glycosylation-related glycogenes in both tissues^49^. We consider that the obvious differences of glycome patterns between different organs may be related to their developmental origins from different germ layers.

In addition to the differential glycomic profiling of various organs, the present study revealed differential glycan expression in morphologically different regions of the kidney, i.e., a higher level of fucosylation in the cortex, and higher levels of sialylation and GlcNAc oligomers in the medulla and pelvis (Fig. 3a–c), which were also confirmed by lectin staining (Fig. 3d). The high level of fucosylation (recognized by AOL, AAL, LCA, PSA, *et al*.) in the cortex is also consistent with previous reports analyzing the *N*-glycome in the kidney by MALDI-IMS, which showed that the following glycans are specifically present in the cortex section: (Hex)_2_(HexNAc)_2_(Deoxyhexose)_3_ + (Man)_3_(GlcNAc)_2_ (*m/z* 2158.76), (Hex)_2_(HexNAc)_3_(Deoxyhexose)_3_ + (Man)_3_(GlcNAc)_2_ (*m/z* 2304.91), and (Hex)_3_(HexNAc)_4_(Deoxyhexose)_4_ + (Man)_3_(GlcNAc)_2_ (*m/z* 2816.12)^28^,^29^.

Moreover, histochemistry using tissue-specific lectins and the subsequent lectin microarray analysis of the tissue fragments dissected based on lectin staining clearly showed that the signals of *O*-glycan binders PNA and WFA were localized at the inner part of the seminiferous tubules, where the spermatocytes are localized (Figs 4 and 5). The relatively high PNA staining observed in the spermatocytes, but not in the spermatogonia that are localized in the outer part, is partly consistent with former studies on the testes of mouse^50^, rat^51^, and lesser mouse-deer^52^. Interestingly, the Tn-antigen, as detected by HPA, is expressed in some seminiferous tubules but not in others (Fig. 4); the differences in HPA staining may be reflected by differences in seminiferous tubules, resulting in little difference between the HPA signals of the inner and outer parts of individual cells (Fig. 5d). A consistent result was obtained by a comparison study in bovine testis; the *O*-glycan patterns of fetal and adult tissue specimens were also different from each other^53^. These observations suggest that the functional roles of *O*-glycans at different stages of spermatogenesis are complex and thus need to be further investigated in detail. Such a relationship between the degree of spermatogenesis and alteration in glycome profile, which comports well with the obvious difference in staining pattern among *O*-glycan binding lectins (HPA, WFA, and PNA) as shown in Fig. 4, will be clearly revealed when differential glycomic analyses are successfully performed among individual tubules using advanced lectin microarray technology with 10-fold higher sensitivity.

In conclusion, we successfully established a simple and reproducible method for rapid and systematic differential glycomic analysis of FFPE tissue sections. Using this approach, we revealed differential glycomic profiles in different organs and in different parts of one organ. The present differential profiling strategy is a highly practical approach for the discovery of physiopathologically relevant alterations in extremely small tissue sections, and thus facilitates disease-related biomarker discovery. Furthermore, as a universal method, this strategy can be easily applied by other researchers and enables new international collaboration studies for high-throughput analysis. This is the first attempt to standardize tissue glycome mapping. Comparing tissue glycome profiles between different organs at different stages is important but difficult. The merit of using a lectin microarray is that it provides a means to rapidly and efficiently obtain initial glycome differences in crude biological samples, and select the proper tissue-specific lectin probes for further clinical analysis. At the same time, several issues emerged that should be solved in the future, such as the cross-reactivity of some lectins, which complicates the interpretation of the observed lectin binding patterns, and the lot-to-lot variation between lectin chips, which may be due to variations during the purification of lectins, the production of the glass, or the spotting process. In addition, as the current 45 lectins on the LecChip account for the most common, but not all possible glycan variants in tissue samples, further in-depth glycomic analyses will require additional lectins that complement the specificities of those on the lectin microarray, e.g., sulfated glycan binding lectins. Once these issues are addressed, our approach will contribute to the construction of the “glycoproteome atlas” under the direction of the Biology/Disease-driven Human Proteome Project Initiative of the Human Proteome Organization (HUPO).

## Materials and Methods

### Animals

All experiments were conducted in accordance with the approval of the Institutional Animal Care and Use Committee at the National Institute of Advanced Industrial Science and Technology, and all methods were performed in accordance with the relevant guidelines and regulations. Two male C57BL/6J mice (named Mouse 2 and 3) were bred and housed in a specific pathogen-free animal facility with access to food and water *ad libitum*. At 8 weeks old, the mice were euthanized by excessive anesthesia with 2-propanol inhalation and the following organs and tissues were collected: brain, liver, kidney, spleen, and testis. A commercialized tissue array consisting of slices of 11 FFPE organ blocks (brain, lung, heart, thymus, spleen, pancreas, skin, kidney, intestine, testis, and liver) from a C57BL/6J mouse (8 weeks, male) made by perfusion fixation with 4% paraformaldehyde for 36 h was purchased from Tsukuba GeneTechnology Laboratories Inc. (Ibaraki, Japan).

### Tissue sample preparation

All the collected mouse tissues were immediately placed in 10% (v/v) formalin prior to processing for routine paraffin embedding. The FFPE tissue samples were sectioned at a thickness of 2 μm and stained with hematoxylin and eosin (H&E) for confirmation of morphology. For LMD, the FFPE tissue sections (5-μm thickness) of Mouse 2 and 3 were mounted on polyethylene naphthalate membrane-coated slides. All the sections were deparaffinized three times in xylene for 10 min each, dehydrated with 100%, 95%, 90%, 80%, and 70% (v/v) ethanol solutions for 5 min each, washed with Milli-Q water, and stained with hematoxylin. The slides were air-dried and stored at room temperature (RT) until use.

### Tissue segmentation

The hematoxylin-stained tissue sections were cut into several small regions depending on different histomorphological features. For the tissue array containing 11 mouse organs, each small region (~1.0 mm^2^) was manually dissected using a 21-G needle under a microscope, and collected into a 1.5-mL microtube containing 200 μL of 10 mM citrate buffer (pH 6.0). On the other hand, the tissue sections of Mouse 2 and 3 were subjected to LMD with a Leica LMD6000 (Leica Microsystems, Wetzlar, Germany). Each microdissected tissue fragment (~0.9 mm^2^) was collected into a 0.5-mL tube and the tubes were centrifuged at 20,000× *g* for 1 min at 4 °C, followed by adding 200 μL of 10 mM citrate buffer (pH 6.0).

### Protein extraction and lectin microarray

Protein extraction and lectin microarray analysis were basically performed as described previously^35^. Briefly, the antigen retrieval procedure was performed by incubating the tubes containing the tissue fragments and buffer at 95 °C for 1 h. After the addition of 4 μL of a 50% slurry of microcrystalline cellulose into the tube and a wash with phosphate-buffered saline (PBS) by centrifugation, the tissue pellets were solubilized with 20 μL of PBS containing 0.5% Nonidet P-40 and sonicated three times for 10 s each. Subsequently, the tissue suspension was incubated on ice for 1 h and centrifuged at 20,000× *g* for 1 min at 4 °C. The obtained supernatants (20 μL) were fluorescence-labeled with 10 μg of Cy3-succinimidyl ester (GE Healthcare, Buckinghamshire, UK) at RT for 1 h in the dark. The sample solution was adjusted to 100 μL with probing buffer (500 mM glycine, 1 mM CaCl_2_, and 1 mM MnCl_2_ in Tris-buffered saline containing 1.0% Triton X-100) and incubated at RT for 2 h to block the excess fluorescent reagent. Each 60 μL aliquot of Cy3-labeled glycoprotein was applied to one well on a lectin microarray chip (LecChip^TM^, GlycoTechnica, Yokohama, Japan) and incubated 16 h at 20 °C. Fluorescence signals were measured using a chip scanner GlycoStation^TM^ Reader 1200 (GlycoTechnica), and the data were analyzed with Array-Pro Analyzer software (Version 4.5, Media Cybernetics, Bethesda, MD, USA). The net intensity was calculated by subtracting the background from the signal intensity. We selected the scanning data under appropriate gain conditions, which provided the net intensities of all positive spots <40,000. Carbohydrate specificities and abbreviations of the 45 lectins on the LecChip are shown in Supplementary Table S1. The relative intensity of each lectin was normalized. In general, four different normalization methods are available for lectin microarray data: “max,” “mean,” “particular lectin,” and “median”^54^,^55^. The principles for selection of a proper normalization method are known as follows. In the case of crude samples such as cell lysates, tissue extracts, and specific proteins with a large number of glycans (like mucin), more lectin signals can be obtained and the pattern does not change drastically. In such cases, the mean normalization is preferred, which give significantly lower average CVs in comparison with other normalized methods^54^. On the other hand, in a case of a specific glycoprotein, it is usually composed of 5 to 20 lectin signals, and the number of differential lectins is limited^56^,^57^,^58^. In such cases, it is preferable to normalize with a specific lectin. Based on these principles, the relative intensity of each lectin was normalized by the mean of signal intensities of all lectins in this study.

### Statistical analysis

Principal component analysis (PCA) and hierarchical clustering (HC) analysis were performed using MATLAB (version 2010a, The Math Works, Natick, MA, USA) and open-source software MeV (Multiple Experiment Viewer, version 4.8.1), respectively, to compare the signal patterns of 45 lectins in different tissue sections. The normalized data were subjected to HC analysis by using the Pearson correlation coefficient as the distance metric and average linkage analysis method. A Mann–Whitney *U* test was used to compare the lectin intensities between different regions of kidney. A *P* value < 0.05 was considered statistically significant.

### Lectin staining of mouse tissues

FFPE tissue sections (2 μm thickness) of mouse kidney and testis were used for lectin staining. After deparaffinization and dehydration through a graded series of ethanol solutions, the tissue sections were transferred to Target Retrieval Solution, Citrate pH 6.0 (S2031; DAKO Corporation, Carpinteria, CA, USA) and autoclaved for 10 min at 110 °C to improve the lectin reactivity. Endogenous biotin activity was quenched with Biotin Blocking system (X0590; DAKO Corporation). After washing twice with PBS, the glass slides were incubated with Carbo-Free Blocking Solution (SP-5040; Vector Laboratories, Burlingame, CA, USA) for 30 min at RT in a humid chamber. For biotinylated lectin staining, the glass slides were incubated with 5 μg/mL of a biotinylated lectin (*Sambucus nigra* agglutinin, SNA; *Helix pomatia* agglutinin, HPA; WFA; *Arachis hypogaea* agglutinin, PNA; and *Galanthus nivalis* agglutinin, GNA; Vector Laboratories) for 16 h at 4 °C in the dark, and subsequently incubated with 1 μg/mL of Streptavidin-Alexa 594 (S32356; Invitrogen, Carlsbad, CA, USA) at 20 °C for 1 h. For fluorescein isothiocyanate (FITC)-labeled lectin staining, the glass slides were directly incubated with 5 μg/mL of a FITC-labeled lectin (*Aleuria aurantia* lectin, AAL; and *Lycopersicon esculentum* lectin, LEL; Vector Laboratories) at 20 °C for 2 h in the dark. Finally, the glass slides were incubated with 0.5 μg/mL of Hoechst 33342 (364-07951; Dojindo, Tokyo, Japan) and sealed with one drop of Prolong Gold (P36930; Invitrogen). Quantitative measurements of the lectin signals were obtained from 4 representative images using Image-Pro Plus (version 6.0, Media Cybernetics, Bethesda, MD, USA).

## Additional Information

**How to cite this article**: Zou, X. *et al*. A standardized method for lectin microarray-based tissue glycome mapping. *Sci. Rep.* **7**, 43560; doi: 10.1038/srep43560 (2017).

**Publisher's note:** Springer Nature remains neutral with regard to jurisdictional claims in published maps and institutional affiliations.

## Supplementary Material

Supplementary Information

Supplementary Dataset 1

## Figures and Tables

**Table 1:** Significant lectin signals in different parts of kidney.

No.	Lectin	Net intensity^a^	Group 1^b^	Group 2^b^	Ratio (Group1/Group2)	*P*^c^
Strong signals in cortex						
5	AOL	13793.48	2.58	1.19	2.17	2.70E-07
6	AAL	16000.00	2.87	1.64	1.75	2.62E-06
23	BPL	3230.43	0.61	0.22	2.75	5.30E-05
3	LCA	6765.22	1.15	0.76	1.51	2.45E-04
2	PSA	5552.17	0.97	0.59	1.62	2.94E-04
40	Calsepa	32247.83	5.28	4.38	1.20	7.09E-04
18	ConA	8789.13	1.43	1.10	1.30	5.49E-03
29	UDA	24263.04	3.85	3.41	1.13	0.05
36	HPA	1254.35	0.22	0.12	1.86	0.05
Weak signals in cortex						
8	SNA	10358.70	1.33	1.87	0.71	7.13E-05
7	MAL	2447.83	0.25	0.52	0.48	7.49E-05
35	MPA	2204.35	0.23	0.45	0.52	1.27E-04
10	TJA-I	14021.74	1.75	2.57	0.68	2.69E-04
9	SSA	9660.87	1.26	1.70	0.74	9.94E-04
27	LEL	21767.39	3.06	3.69	0.83	5.49E-03
31	Jacalin	9991.30	1.40	1.59	0.88	0.01
22	TxLC-I	2163.04	0.21	0.44	0.49	0.01
15	DSA	23089.13	3.12	3.90	0.80	0.02
14	PHA-E	7945.65	1.08	1.31	0.82	0.02
21	ACG	7867.39	1.02	1.48	0.69	0.04
24	TJA-II	2821.74	0.33	0.51	0.65	0.05

^a^The average net intensity of lectin in kidney samples.

^b^Group 1, the average normalized intensity of lectin in cortex parts; Group 2, the average normalized intensity of lectin in glomerulus, medulla, and pelvis parts.

^c^*P*-value of Mann-Whitney *U* test.
