# Supplementary material for: A standardized method for lectin microarray-based tissue glycome mapping
Source: Sci Rep. 2017 Mar 6;7:43560. doi: 10.1038/srep43560 (PMC5337905; doi:10.1038/srep43560)
Supplement: Supplementary Information [file srep43560-s1.pdf]

A standardized method for lectin microarray-based tissue glycome mapping

Xia Zou[1,2,#], Maki Yoshida[1,#], Chiaki Nagai-Okatani[1,#], Jun Iwaki[1], Atsushi Matsuda[1], Binbin Tan[1,2], Kozue Hagiwara[1], Takashi Sato[1], Yoko Itakura[3], Erika Noro[1], Hiroyuki Kaji[1], Masashi Toyoda[3], Yan Zhang[2], Hisashi Narimatsu[1], Atsushi Kuno[1,*]

Supplementary Figure legends

Figure S1. Differential glycomic profiling of tissue fragments obtained by manual dissection. (a) Tissue section images of 11 mouse organs on a commercialized tissue array. A total of 94 tissue fragments (15 fragments of brain, 6 of heart, 7 of liver, 10 of kidney, 4 of lung, 4 of pancreas, 10 of skin, 13 of small intestine, 7 of spleen, 12 of testis, and 6 of thymus) were obtained by dissection by hand. (b) Two-dimensional HC analysis for the 94 tissue fragments based on normalized data of the lectin microarray. The 94 samples are listed in columns and the 45 lectins are listed in rows. The color and intensity of each square indicate the lectin signal levels in specific tissue fragment. Red, high; green, low; black, medium.

Figure S2. Comparison of the scanning gains between manual dissection (a) and laser microdissection (b). The scanning gains were optimized as the net intensities of all positive spots < 40,000 for each tissue fragment of brain (upper panel) and testis (lower panel), respectively.

Figure S3. Representative images of tissue dissection by LMD. Tissue fragments were collected from tissue sections of the five organs (brain, liver, kidney, spleen, and testis) depending on the morphological difference.

Figure S4. Representative images of lectin profiling in five organs from Mouse 2.

Figure S5. Representative glycomic profiles in five organs. The normalized lectin microarray data corresponding to tissue fragments from five organs (19 fragments of brain, 6 of liver, 13 of kidney, 4 of spleen, and 6 of testis) shown in Figure S3 are indicated.

Figure S6. Sample inclusion criteria for statistical analysis in this study. A total of 190 tissue fragments from two mice were collected, and 7 samples (2 samples of brain and 5 samples of kidney) with low signal intensity that were scanned under the appropriate gain conditions of 105 and 115, and 1 sample of testis with a high noise level were excluded from the subsequent statistical analysis. The appropriate gain conditions of the remaining 182 samples were between 65 and 85.

Figure S7. Differential *N*- and *O*-glycomic profiling in the 182 tissue fragments. PCA was performed using the 182 samples based on the normalized signals of 33 lectins recognizing *N*-glycans (a), and 32 lectins recognizing *O*-glycans (b), respectively. Each point represents one tissue fragment. Two-dimensional HC analysis was also performed using the normalized data for *N*-glycans (c) and *O*-glycans (d), respectively. The 182 samples are listed in columns and the corresponding 33 or 32 lectins are listed in rows. The color and intensity of each square indicate the lectin signal levels in specific tissue fragments (Red, high; green, low; and black, medium).

Figure S8. Differential glycomic profiling in kidney from Mouse 2 (a) and Mouse 3 (b and c). Left panels show PCA of the normalized lectin microarray data on the tissue fragments

obtained from kidney sections. Each point represents one tissue section. Right panels show two-dimensional HC analysis of the normalized data. The samples are listed in columns and the 45 lectins are listed in rows. The color and intensity of each square indicate the expression levels of specific lectin signal levels in specific tissue fragments (Red, high; green, low; and black, medium).

Figure S9. Quantitative measurements of lectin signals for AAL (a), SNA (b), and LEL (c) staining shown in Figure 3d and corresponding lectin microarray analysis in renal cortex and medulla. Quantitative data of lectin staining were obtained from 4 representative images using Image-Pro Plus. Data are shown as means ±SD.

Figure S10. Representative images of tissue dissection from the total, inner, and outer parts of seminiferous tubules. Each fragment was collected from 30 seminiferous tubules by LMD; the inner and outer parts were collected from identical tubules.

Supplementary Table 1. Binding specificities, origin, and abbreviations of 45 lectins on the LecChip.

No.	Lectins	Origin	Binding specificity
1	LTL	*Lotus tetragonolobus*	Fucα1-3GlcNAc, Sia-Le^x and Le^x
2	PSA	*Pisum sativum*	Fucα1-6GlcNAc and α-Man
3	LCA	*Lens culinaris*	Fucα1-6GlcNAc, α-Man and α-Glc
4	UEA-I	*Ulex europaeus*	Fucα1-2(Galβ1-4)GlcNAc
5	AOL	*Aspergillus oryzae*	Fucα1-6GlcNAc and Fucα1-2(Gal β1-4)GlcNAc
6	AAL	*Aleuria aurantia*	Fucα1-6GlcNAc and Le^x
7	MAL	*Maackia amurensis*	Siaα2-3Galβ1-4GlcNAc
8	SNA	*Sambucus nigra*	Siaα2-6Gal/GalNAc
9	SSA	*Sambucus sieboldiana*	Siaα2-6Gal/GalNAc
10	TJA-I	*Trichosanthes japonica*	Siaα2-6Gal/GalNAc and Gal
11	PHA-L	*Phaseolus vulgaris*	Tri- and tetra-antennary complex-type *N*-glycans
12	ECA	*Erythrina cristagalli*	Galβ1-4GlcNAc
13	RCA120	*Ricinus communis*	Galβ1-4GlcNAc
14	PHA-E	*Phaseolus vulgaris*	Bisecting GlcNAc and biantennary *N*-glycans
15	DSA	*Datura stramonium*	$(GlcNAc\beta1\text{-}4)_n$, polyLacNAc and branched LacNAc

16	GSL-II	*Griffonia simplicifolia*	Agalactosylated *N*-glycan and GlcNAc
17	NPA	*Narcissus pseudonarcissus*	non-substituted α1-6Man
18	ConA	*Canavalia ensiformis*	α-Man (inhibited by presence of bisecting GlcNAc)
19	GNA	*Galanthus nivalis*	non-substituted α1-6Man
20	HHL	*Hippeastrum hybrid*	non-substituted α1-6Man
21	ACG	*Agrocybe cylindracea*	Siaα2-3Gal β1-4GlcNAc
22	TxLC-I	*Tulipa gesneriana*	Man_3 core, bi- and tri-antennary complex-type *N*-glycan and GalNAc
23	BPL	*Bauhinia purpurea alba*	Galβ1-3GalNAc and GalNAc
24	TJA-II	*Trichosanthes japonica*	β-GalNAc and Fucα1-2Gal
25	EEL	*Euonymus europaeus*	Galα1-3(Fuc α1-2)Gal
26	ABA	*Agaricus bisporus*	Gal, Galβ1-3GalNAc and sialyl-T
27	LEL	*Lycopersicon esculentum*	$(GlcNAc)_n$ and polyLacNAc
28	STL	*Solanum tuberosum*	$(GlcNAc)_n$ and polyLacNAc
29	UDA	*Urtica dioica*	$(GlcNAc)_n$ and polyLacNAc
30	PWM	*Phytolacca americana*	$(GlcNAc)_n$ and polyLacNAc
31	Jacalin	*Artocarpus integrifolia*	Galβ1-3GalNAcα-Thr/Ser (T) and GalNAcα-Thr/Ser (Tn)
32	PNA	*Arachis hypogaea*	Galβ1-3GalNAcα-Thr/Ser (T)
33	WFA	*Wisteria floribunda*	Terminal GalNAc (e.g.,

			GalNAcβ1-4GlcNAc) and Galβ1-3(-6)GalNAc
34	ACA	*Amaranthus caudatus*	Galβ1-3GalNAcα-Thr/Ser (T)
35	MPA	*Maclura pomifera*	Galβ1-3GalNAcα-Thr/Ser (T) and GalNAcα-Thr/Ser (Tn)
36	HPA	*Helix pomatia*	α-linked terminal GalNAc
37	VVA	*Vicia villosa*	GalNAcα-Thr/Ser (Tn) and GalNAcα1-3 Gal
38	DBA	*Dolichos biflorus*	GalNAcα-Thr/Ser (Tn) and GalNAcα1-3GalNAc
39	SBA	*Glycine max*	Terminal GalNAc (especially GalNAcα1-3Gal)
40	Calsepa	*Calystegia sepium*	High Man and *N*-glycans including bisecting GalNAc
41	PTL-I	*Psophocarpus tetragonolobus*	α-GalNAc and Gal
42	MAH	*Maackia amurensis*	Siaα2-3Gal β1-3(Sia α2-6) GalNAc
43	WGA	*Triticum unlgaris*	(GlcNAc)n and multivalent Sia
44	GSL-IA_4	*Griffonia simplicifolia*	α-GalNAc and GalNAcα-Thr/Ser (Tn)
45	GSL-IB_4	*Griffonia simplicifolia*	α-Gal

Supplementary Table 3. Classification based on the binding specificities of the 45 lectins on the LecChip.

Classification	Number	Name of Lectins
Lectins recognizing *N*-glycans	13	LTL; PSA; LCA; PHA-L; PHA-E; GSL-II; TxLC-I; NPA; ConA; GNA; HHL; Calsepa; UDA;
Lectins recognizing *O*-glycans	12	MAH; GSL-IB4; Jacalin; VVA; MPA; GSL-IA4; PNA; ACA; ABA; SBA; DBA; PTL-I
Lectins recognizing both *N*-glycans and *O*-glycans	20	AOL; AAL; UEA-I; MAL; SNA; SSA; TJA-I; ACG; ECA; RCA120; TJA-II; EEL; DSA; LEL; STL; PWM; WGA; BPL; WFA; HPA

Fig. S1

a

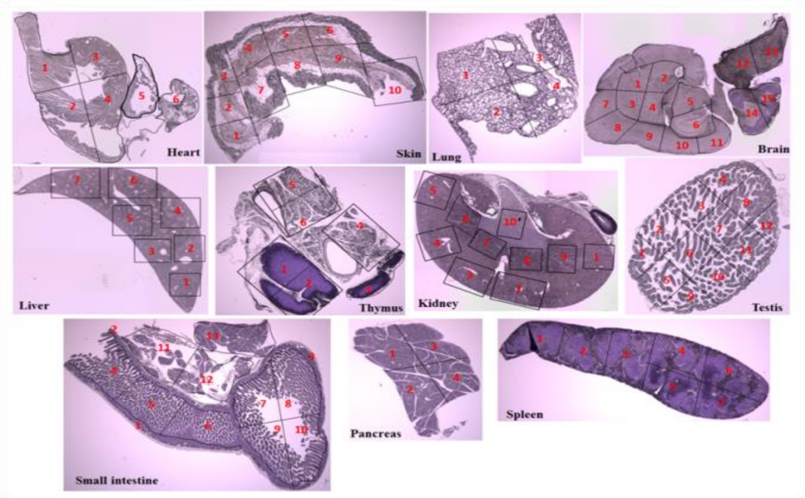
Heart
Skin
Lung
Brain
Liver
Thymus
Kidney
Testis
Small intestine
Pancreas
Spleen

b

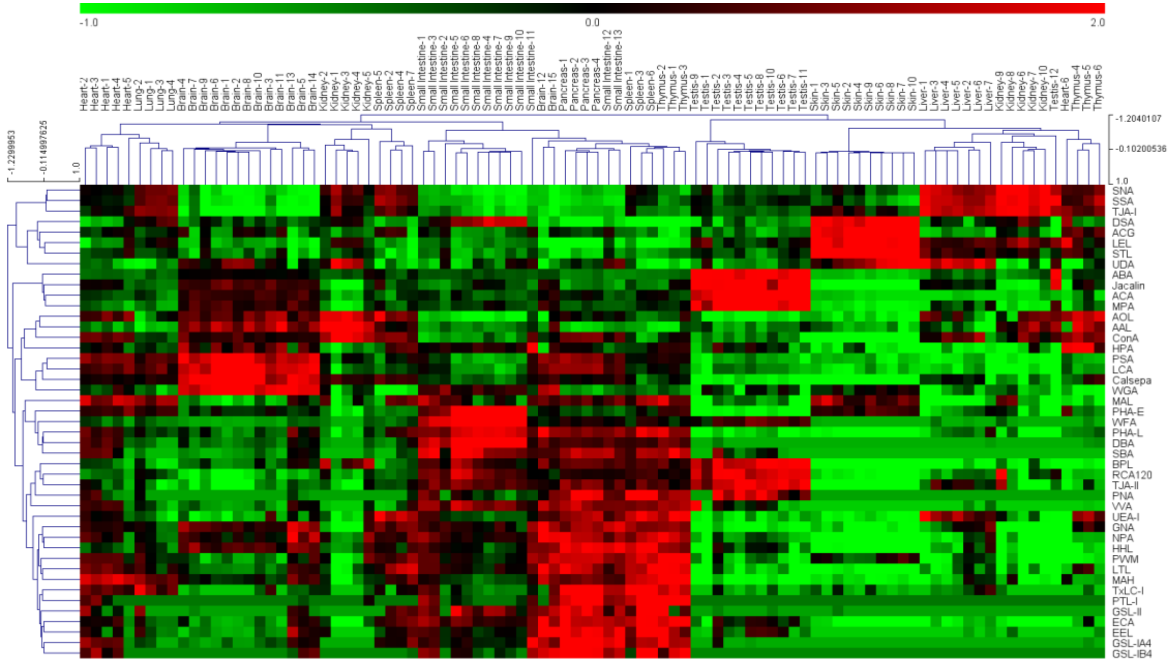
-1.0
0.0
2.0
-1.2040107
-0.10200536
1.0
-1.2299953
-0.114997625
1.0
SNA
SSA
TJA-I
DSA
ACG
LEL
STL
UDA
ABA
Jacalin
ACA
MPA
AOL
AAL
ConA
HPA
PSA
LCA
Calsepa
WGA
MAL
PHA-E
WFA
PHA-L
DBA
SBA
BPL
RCA120
TJA-II
PNA
VVA
UEA-I
GNA
NPA
HHL
PWM
LTL
MAH
TxLC-I
PTL-I
GSL-II
ECA
EEL
GSL-IA4
GSL-IB4

Fig.S2

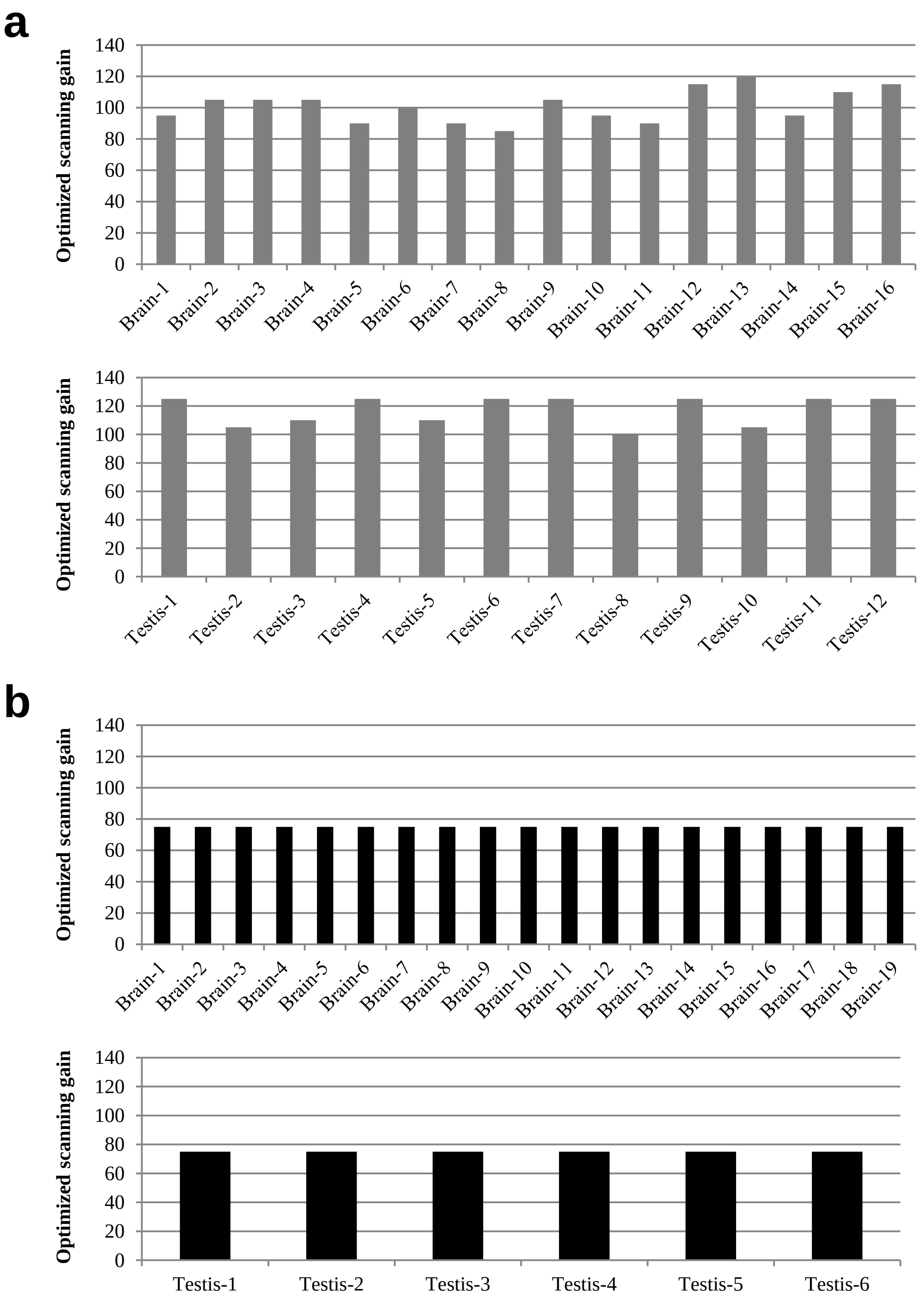
a
b
Optimized scanning gain
0
20
40
60
80
100
120
140
Brain-1
Brain-2
Brain-3
Brain-4
Brain-5
Brain-6
Brain-7
Brain-8
Brain-9
Brain-10
Brain-11
Brain-12
Brain-13
Brain-14
Brain-15
Brain-16
Brain-17
Brain-18
Brain-19
Testis-1
Testis-2
Testis-3
Testis-4
Testis-5
Testis-6
Testis-7
Testis-8
Testis-9
Testis-10
Testis-11
Testis-12

Fig.S3

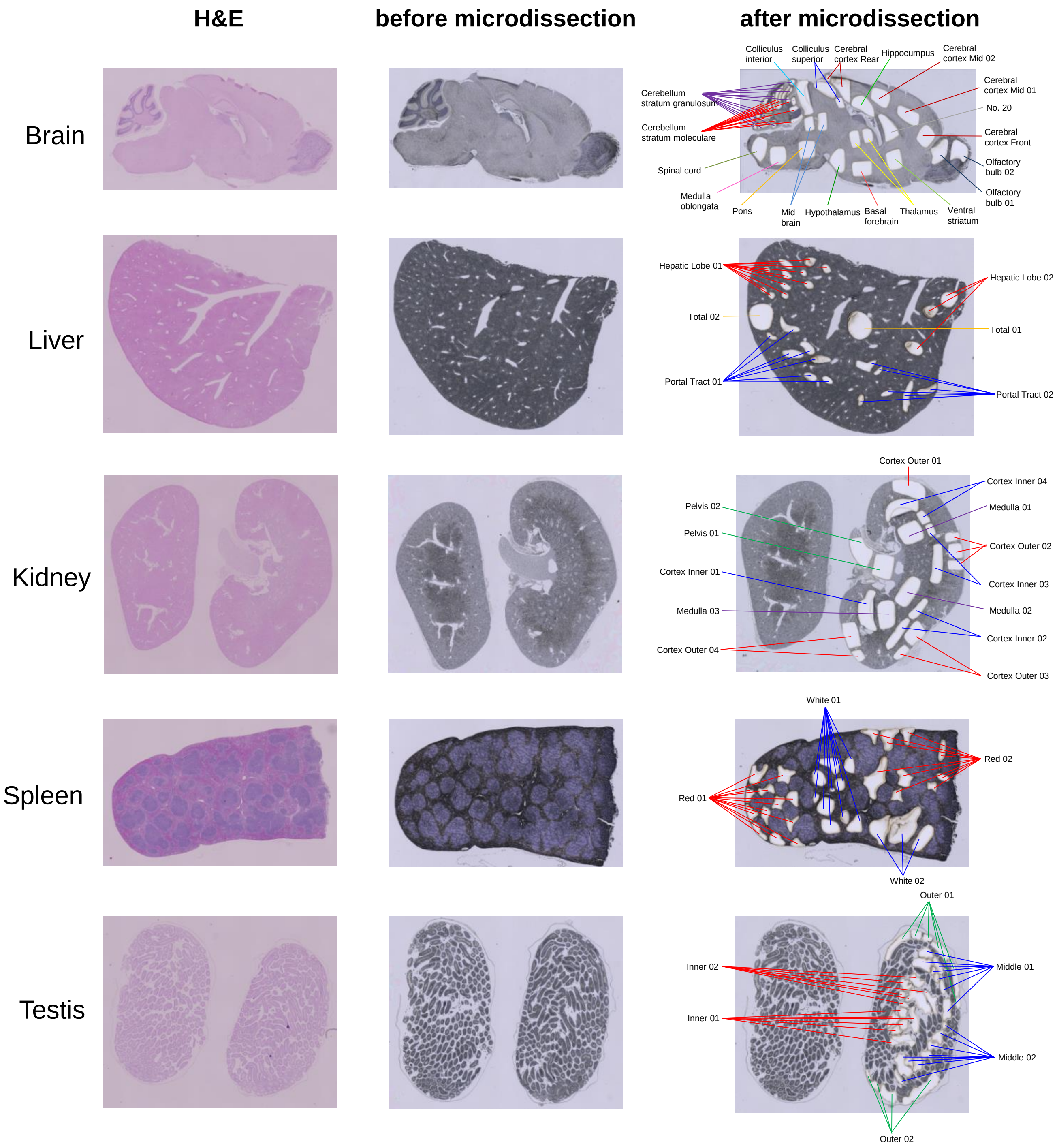
H&E
before microdissection
after microdissection
Brain
Colliculus interior
Colliculus superior
Cerebral cortex Rear
Hippocumpus
Cerebral cortex Mid 02
Cerebral cortex Mid 01
No. 20
Cerebral cortex Front
Olfactory bulb 02
Olfactory bulb 01
Cerebellum stratum granulosum
Cerebellum stratum moleculare
Spinal cord
Medulla oblongata
Pons
Mid brain
Hypothalamus
Basal forebrain
Thalamus
Ventral striatum
Liver
Hepatic Lobe 01
Hepatic Lobe 02
Total 02
Total 01
Portal Tract 01
Portal Tract 02
Kidney
Cortex Outer 01
Cortex Inner 04
Medulla 01
Pelvis 02
Pelvis 01
Cortex Outer 02
Cortex Inner 01
Cortex Inner 03
Medulla 03
Medulla 02
Cortex Inner 02
Cortex Outer 04
Cortex Outer 03
Spleen
White 01
Red 02
Red 01
White 02
Testis
Outer 01
Middle 01
Inner 02
Inner 01
Middle 02
Outer 02

Fig.S4

Brain
(Gain 75)

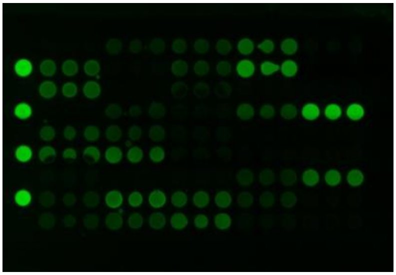

Kidney
(Gain 75)

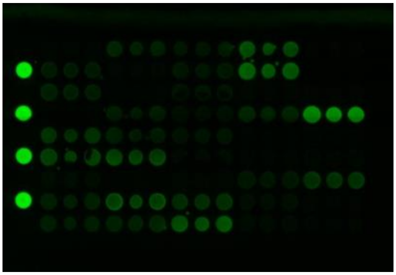

Liver
(Gain 75)

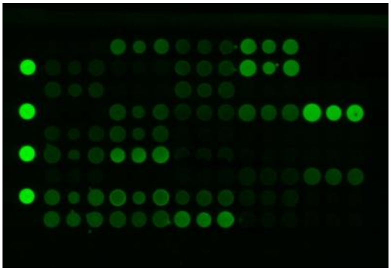

Spleen
(Gain 75)

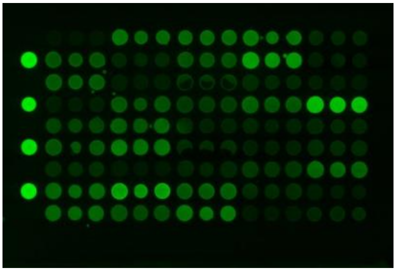

Testis
(Gain 65)

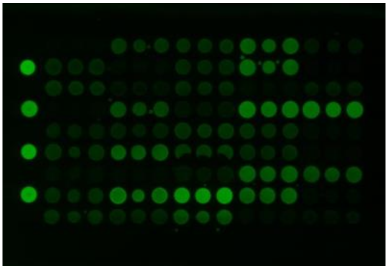

Fig.S5

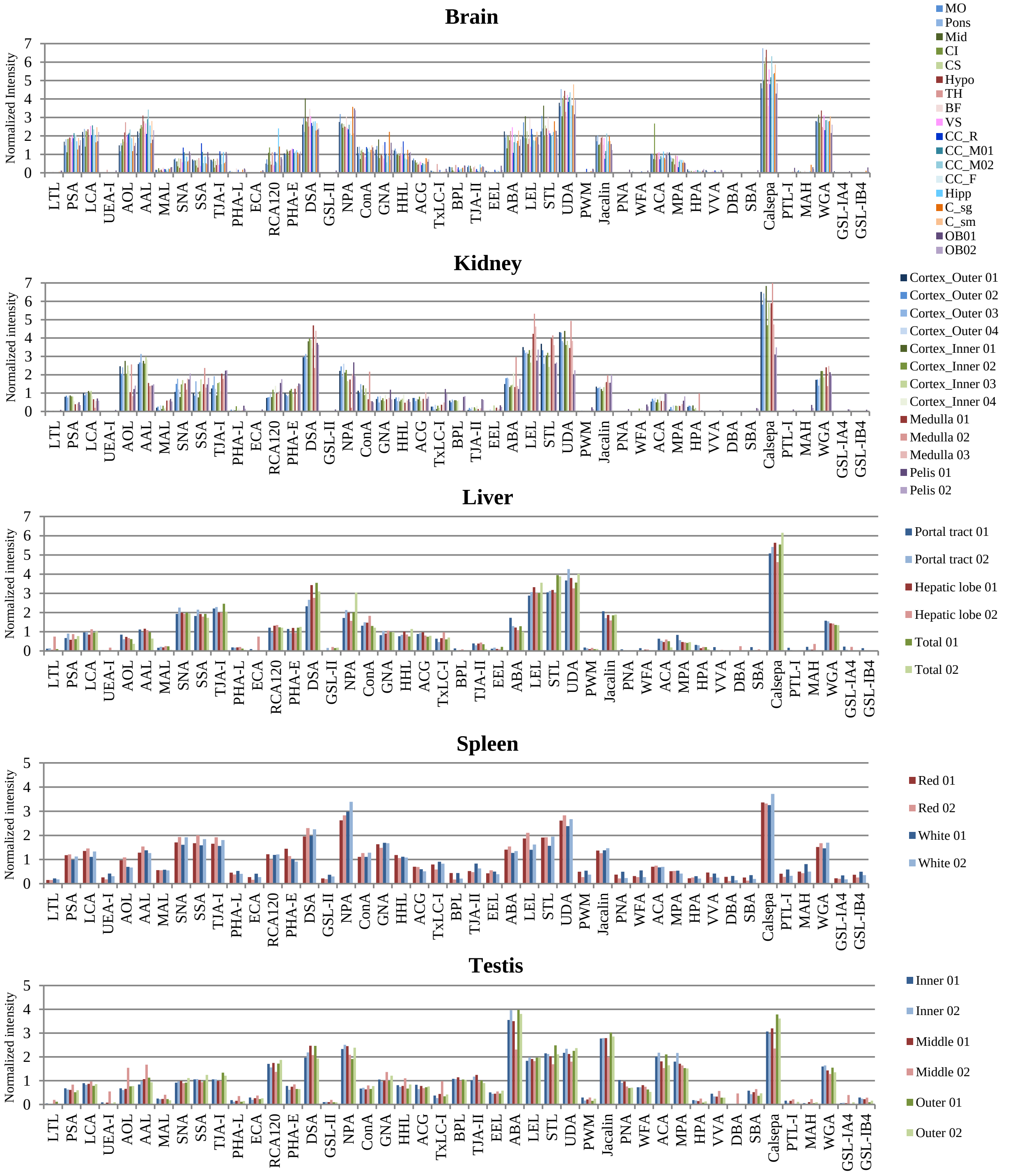
Brain
Normalized Intensity
LTL
PSA
LCA
UEA-I
AOL
AAL
MAL
SNA
SSA
TJA-I
PHA-L
ECA
RCA120
PHA-E
DSA
GSL-II
NPA
ConA
GNA
HHL
ACG
TxLC-I
BPL
TJA-II
EEL
ABA
LEL
STL
UDA
PWM
Jacalin
PNA
WFA
ACA
MPA
HPA
VVA
DBA
SBA
Calsepa
PTL-I
MAH
WGA
GSL-IA4
GSL-IB4
SC
MO
Pons
Mid
CI
CS
Hypo
TH
BF
VS
CC_R
CC_M01
CC_M02
CC_F
Hipp
C_sg
C_sm
OB01
OB02
Kidney
Normalized intensity
Cortex_Outer 01
Cortex_Outer 02
Cortex_Outer 03
Cortex_Outer 04
Cortex_Inner 01
Cortex_Inner 02
Cortex_Inner 03
Cortex_Inner 04
Medulla 01
Medulla 02
Medulla 03
Pelis 01
Pelis 02
Liver
Normalized intensity
Portal tract 01
Portal tract 02
Hepatic lobe 01
Hepatic lobe 02
Total 01
Total 02
Spleen
Normalized intensity
Red 01
Red 02
White 01
White 02
Testis
Normalized intensity
Inner 01
Inner 02
Middle 01
Middle 02
Outer 01
Outer 02

Fig.S6

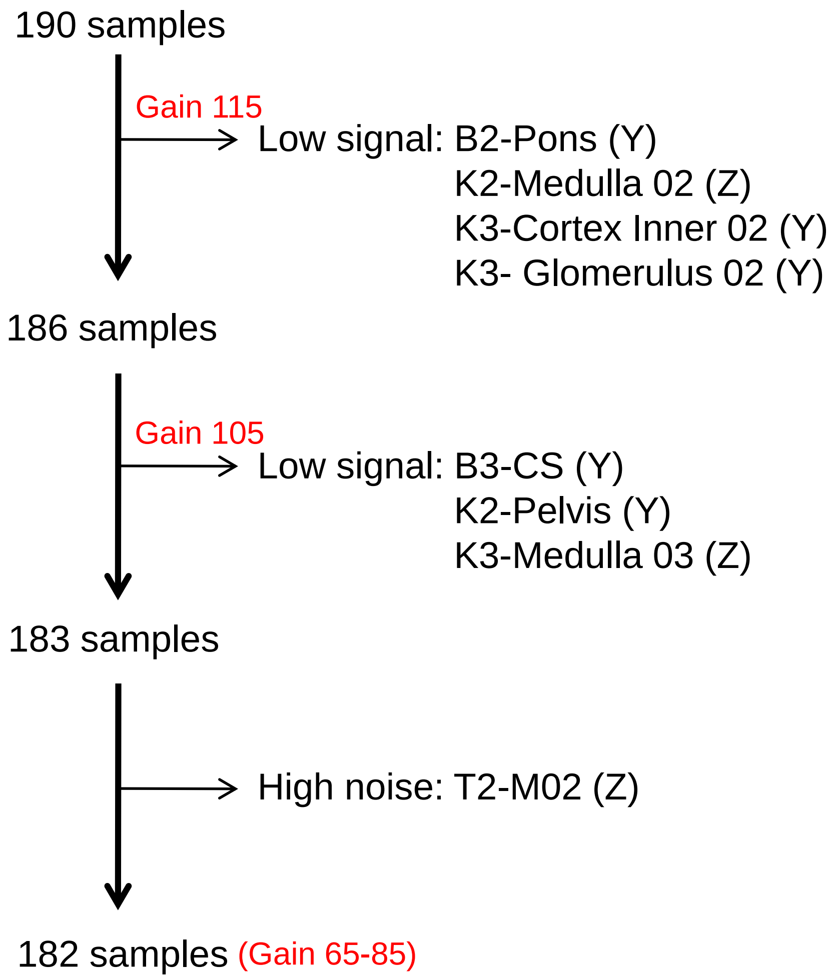
190 samples
Gain 115
Low signal: B2-Pons (Y)
K2-Medulla 02 (Z)
K3-Cortex Inner 02 (Y)
K3- Glomerulus 02 (Y)
186 samples
Gain 105
Low signal: B3-CS (Y)
K2-Pelvis (Y)
K3-Medulla 03 (Z)
183 samples
High noise: T2-M02 (Z)
182 samples (Gain 65-85)

Fig.S7

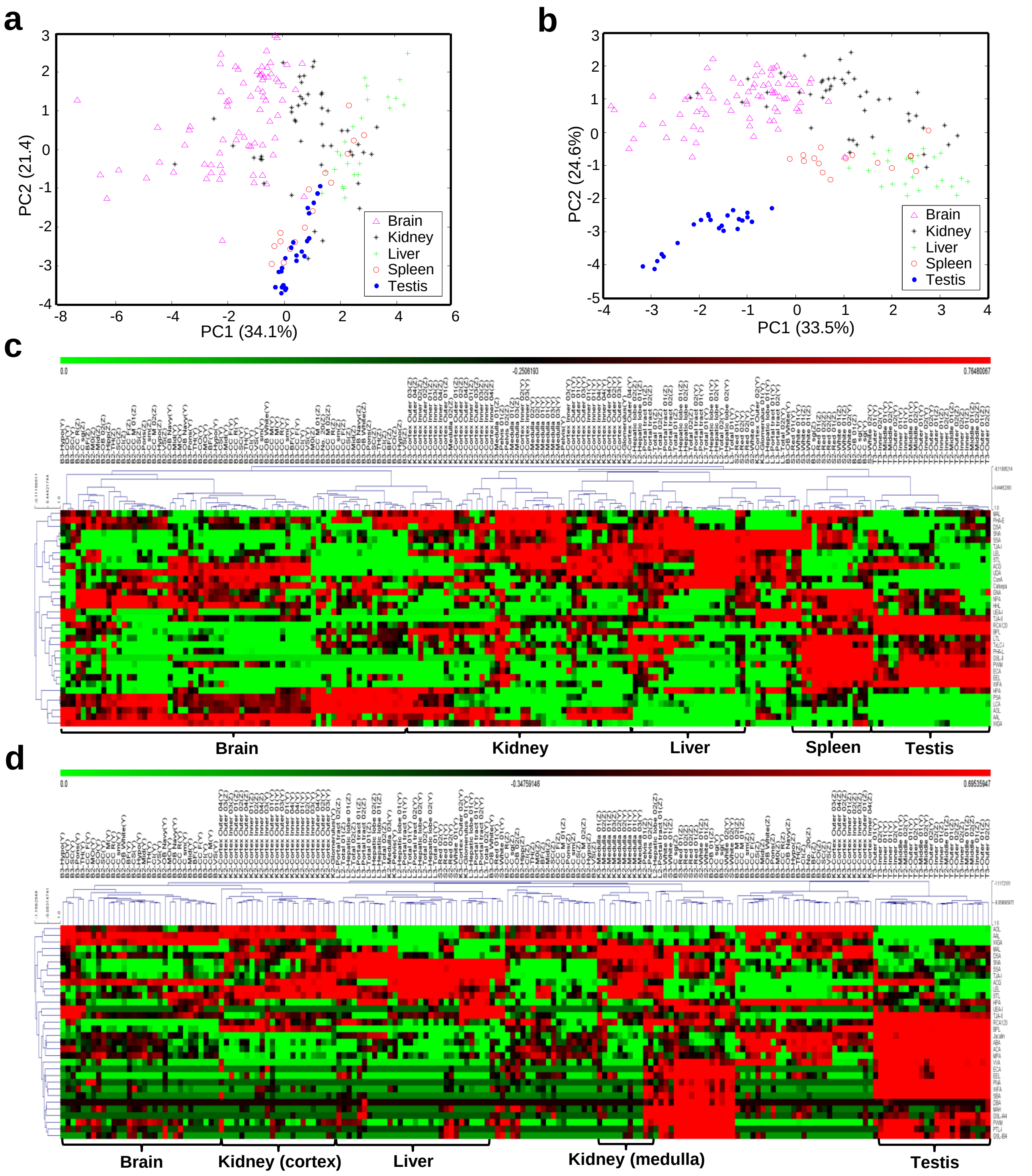
a
b
c
d
PC1 (34.1%)
PC2 (21.4)
PC1 (33.5%)
PC2 (24.6%)
Brain
Kidney
Liver
Spleen
Testis
Brain
Kidney
Liver
Spleen
Testis
Brain
Kidney (cortex)
Liver
Kidney (medulla)
Testis

Fig.S8

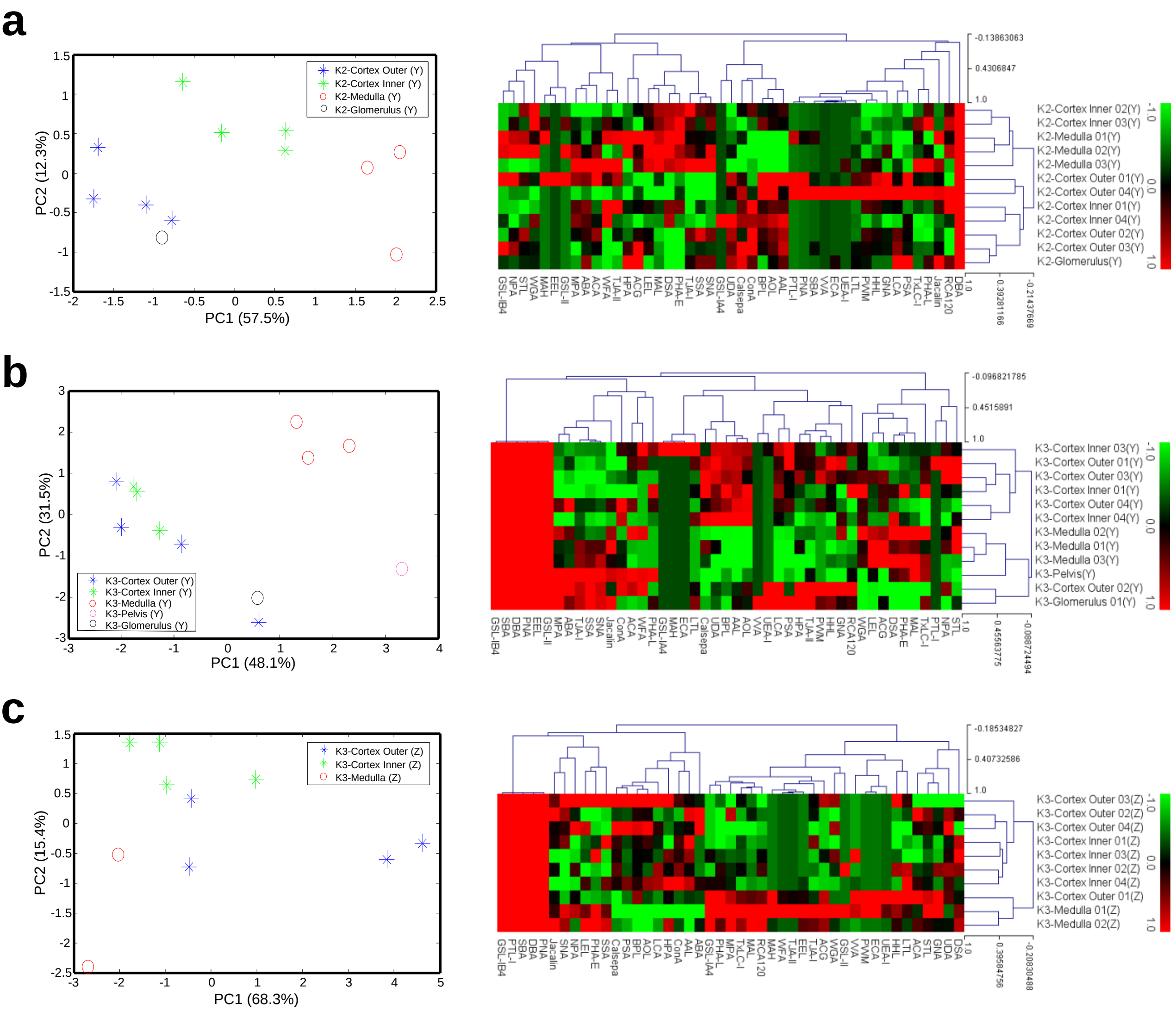
a
b
c
PC1 (57.5%)
PC2 (12.3%)
K2-Cortex Outer (Y)
K2-Cortex Inner (Y)
K2-Medulla (Y)
K2-Glomerulus (Y)
PC1 (48.1%)
PC2 (31.5%)
K3-Cortex Outer (Y)
K3-Cortex Inner (Y)
K3-Medulla (Y)
K3-Pelvis (Y)
K3-Glomerulus (Y)
PC1 (68.3%)
PC2 (15.4%)
K3-Cortex Outer (Z)
K3-Cortex Inner (Z)
K3-Medulla (Z)

Fig.S9

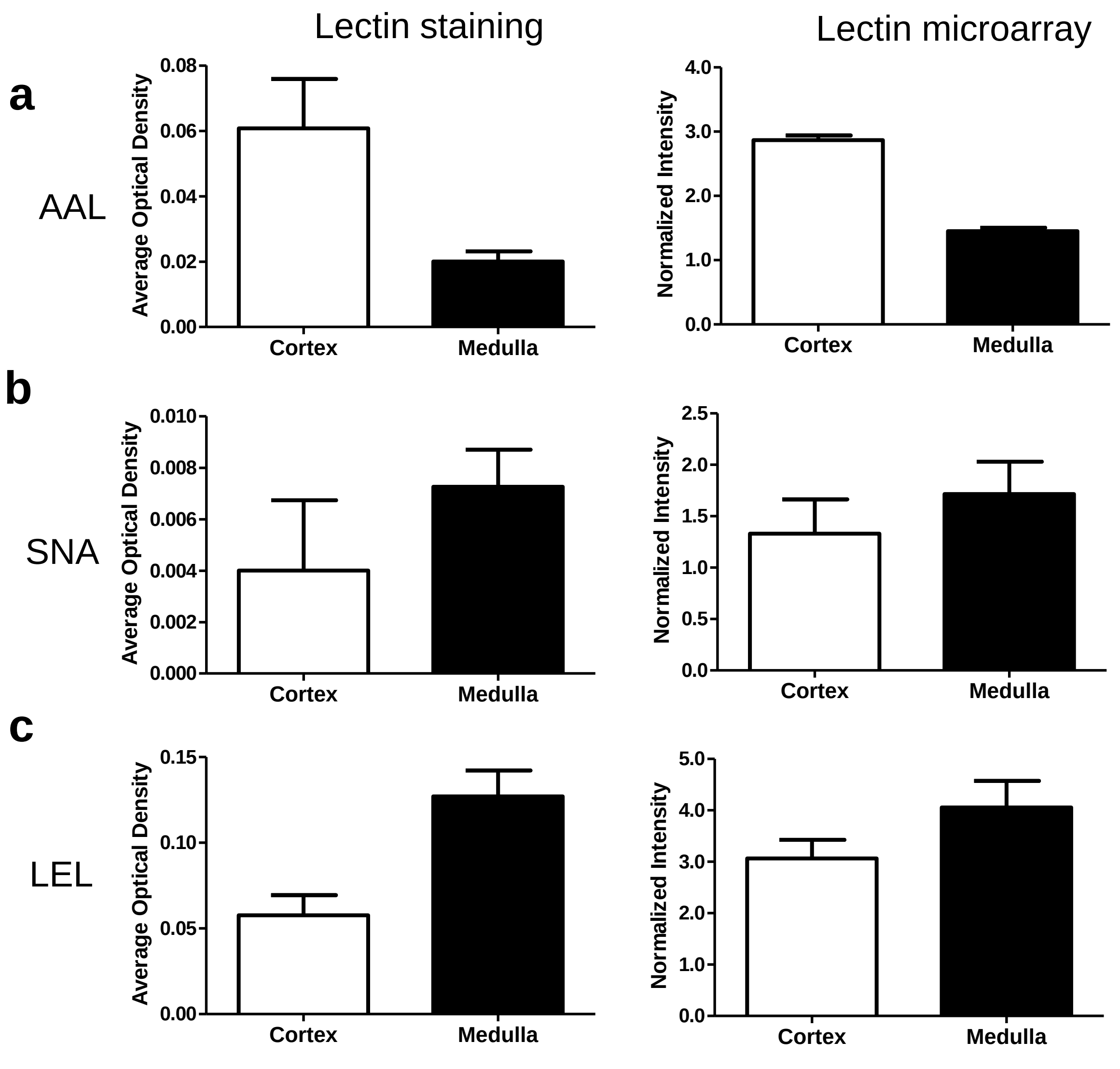
Lectin staining
Lectin microarray
a
AAL
b
SNA
c
LEL
Average Optical Density
Normalized Intensity
Cortex
Medulla
0.00
0.02
0.04
0.06
0.08
0.0
1.0
2.0
3.0
4.0
0.000
0.002
0.004
0.006
0.008
0.010
0.5
1.5
2.0
2.5
0.05
0.10
0.15
5.0

Fig.S10

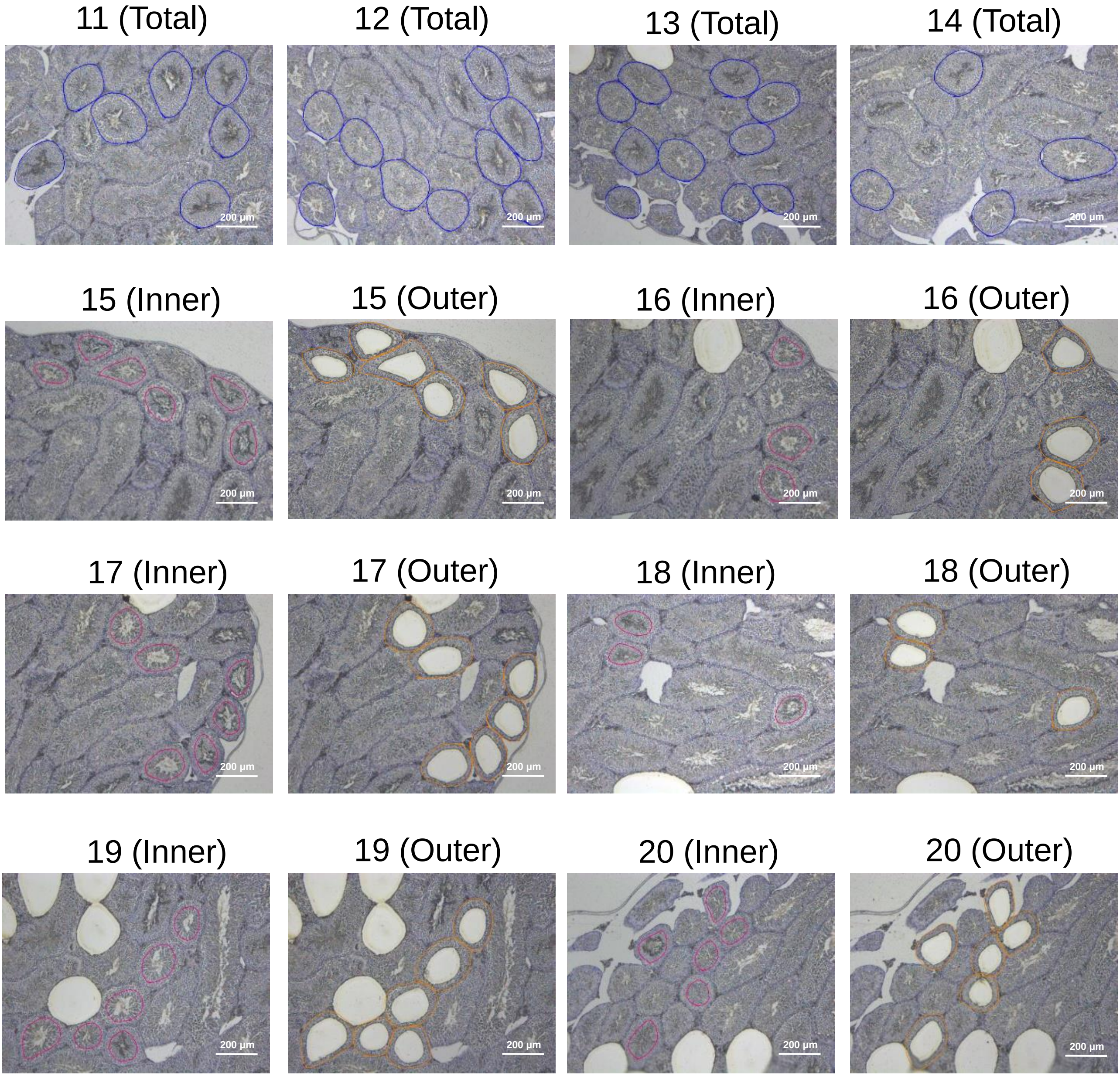
11 (Total)
12 (Total)
13 (Total)
14 (Total)
15 (Inner)
15 (Outer)
16 (Inner)
16 (Outer)
17 (Inner)
17 (Outer)
18 (Inner)
18 (Outer)
19 (Inner)
19 (Outer)
20 (Inner)
20 (Outer)
200 μm
